# Optimizing the growth conditions of superconducting MoSi thin films for single photon detection

**DOI:** 10.1038/s41598-025-86303-5

**Published:** 2025-01-19

**Authors:** Stefanie Grotowski, Lucio Zugliani, Björn Jonas, Rasmus Flaschmann, Christian Schmid, Stefan Strohauer, Fabian Wietschorke, Niklas Bruckmoser, Manuel Müller, Matthias Althammer, Rudolf Gross, Kai Müller, Jonathan Finley

**Affiliations:** 1 Walter Schottky Institute, Technical University of Munich, 85748 Garching, Germany; 2 TUM School of Natural Sciences, Technical University of Munich, 85748 Garching, Germany; 3 TUM School of Computation, Information and Technology, Technicial University of Munich, 85748 Garching, Germany; 4 Walther-Meißner-Institute, Bavarian Academy of Sciences and Humanities, 85748 Garching, Germany; 5 Munich Center for Quantum Science and Technology (MCQST), 80799 Munich, Germany

**Keywords:** Structural properties, Superconducting devices, Superconducting properties and materials

## Abstract

We investigate the growth of amorphous MoSi thin films using magnetron co-sputtering and optimize the growth conditions with respect to crystal structure and superconducting properties (e.g., critical temperature $T_{\text {c}}$). The deposition pressure, Mo:Si stoichiometry and substrate temperature are systematically varied to achieve a transition temperature of 8.4(3) K for films with a thickness of 17.7(8) nm and 6.2(9) K for a 4.3(4) nm thick film. For Mo concentrations above 81% the crystalline phase $\hbox {Mo}_\text {3}$Si is observed in grazing incidence X-ray diffraction measurements. The same phase appears when the working pressure during deposition is reduced below 3.$1 \times 10^{\text {-3}}\hspace{1.66656pt}$mbar and when the substrate temperature during deposition is increased above $100\hspace{1.66656pt}^{\circ }$C. By choosing a sufficient Si concentration and optimum deposition pressure we identify deposition conditions that ensure a homogeneous amorphous growth of the superconducting thin film. We then fabricate superconducting nanowire single-photon detectors which exhibit an unitary internal efficiency to single photons at an operational temperature of 1.2 K while simultaneously having a dark count rate below 1 Hz. Our results establish the link between MoSi film deposition, morphology and the performance of SSPD.

## Introduction

Superconducting nanowire single-photon detectors (SNSPDs) are important for realizing a wide range of classical and quantum photonic technologies. They outperform other single-photon detectors such as single-photon avalanche diodes (SPADs)^1^by exhibiting near unity detection efficiencies in the near infrared (IR) spectral range^2,3^, sub Hz dark count rates^4^, and few picosecond timing jitter^5,6^. At the same time, they must be operated at cryogenic temperatures in order to provide optimum photon detection metrics. Typical applications for SNSPDs are photon-based quantum computing^7,8^, and communications^9,10^, as well as deep space optical communication^11 ^and time of flight measurements^12–14 ^in LIDAR systems^15,16^. The most widely explored thin film superconductors used for SNSPDs are NbN or NbTiN. Both materials have a relatively high transition temperature in the range of $14-$16 K for bulk materials, and $7-$9 K for few nanometer thick films needed for SNSPDs^17,18^. In recent years, amorphous superconductors such as MoSi or WSi have drawn significant attention since they promise enhanced sensitivity in the IR spectral range due to their smaller superconducting energy gap^19^. Both MoSi and WSi typically have critical temperatures close to 7.5 K and 5 K, respectively^20^. Moreover, in contrast to NbN or NbTiN, which are polycrystalline superconductors where the superconducting properties are influenced by the choice of substrate^21^, these amorphous materials promise improved homogeneity and yield in the nanofabricated structures^22–24^. Previous works have also shown that MoSi is particularly suitable for IR single photon detection^25–27^, which is a desired wavelength for the applications mentioned above as the transmission losses in optical fibers are minimized^28^. A crucial factor that governs the resulting performance of SNSPDs is the material quality of the superconducting thin film which can be controlled during the deposition of the superconductor^18,29^.

Motivated by the works of Bosworth *et al.*^30^ and Lita *et al.*^31^ where a relation between crystalline phase formation and superconducting thin film properties were investigated, we systematically investigated the growth conditions of MoSi thin films parameterized against their superconducting properties and their suitability for realizing SNSPDs. Specifically, we control deposition parameters such as pressure, substrate temperature and Mo:Si stoichiometry. By combining electrical transport characterization and grazing incidence X-ray diffraction (GID) measurements we assess the quality of the films and establish structure-property relationships. We optimize the material deposition for films with respect to their superconducting transition temperature ($T_{\text {c}}$) since this defines the maximum bias current that can be applied^3^, thereby improving the sensitivity and timing properties of the SNSPD^32,33^. This opens up the possibility of operating the detector at bias currents far below the switching current, thus reducing the dark count rate (DCR) while maintaining high sensitivity. In addition, the DCR of SNSPDs is known to reduce for operating temperatures well below $T_{\text {c}}$^34^.

## Deposition, nanofabrication and measurement

The superconducting MoSi thin films are deposited by magnetron co-sputter deposition in an ultra-high vacuum (UHV) chamber. Two individual targets of molybdenum (Mo) and silicon (Si) are operated by applying a constant DC current or constant RF power, respectively, igniting a plasma in an argon (Ar) environment. For the co-sputter application the 2 inch targets are tilted by $20^\circ$ towards another and the substrate is placed at 100 mm distance from the targets. By varying the applied current/power, the deposition rates of the materials are controlled individually. Furthermore, the chamber pressure during deposition (working pressure) can be controlled, resulting in an effective change of both deposition rates as the background pressure leads to stronger thermalization of the sputtered atoms, prior to their deposition on the substrate. In addition, the deposition temperature can be adjusted by a heater placed on the backside of the sample. The thickness of the thin film is controlled via the deposition rate measured by a quartz crystal rate sensor and afterwards confirmed by X-ray reflectivity (XRR) measurements. Unless stated otherwise, the films studied in the following have a thickness of 17.7(8) nm and are deposited at room temperature and a working pressure of ${1.2\times {10}^{-2}}\,{\textrm{mbar}}$. This thickness is chosen in order to be close to the bulk materials, where small variations of thickness have a smaller influence on transition temperature $T_{\text {c}}$^35^. We characterize the films by measuring $T_{\text {c}}$ of the samples either in a liquid helium cryostat ($T_{\text {c}}$ > 4.2 K), or in a closed cycle pulse tube cryostat with additional adiabatic demagnetization cooling to reach temperatures down to 0.8 K. After deposition, we analyze the structural phase formation in the material using grazing incidence X-ray diffraction (GID) measurements and the material composition by X-ray photoelectron spectroscopy (XPS).

Subsequently, we fabricate SNSPDs using electron beam lithography for pattering the meander-shaped structure and reactive ion etching for pattern transfer into the film. An electrical connection is established via optical lithography and evaporation of Ti/Au contacts. Finally, the detectors are characterized in a dipstick cryostat connected to a He pump to achieve temperatures down to 1.2 K and the detectors are illuminated with a 780 nm diode laser.

## Results

We begin by exploring the influence of the Mo:Si stoichiometry, the working pressure and substrate temperature used during magnetron co-sputtering to the superconducting properties of the thin films. Hereby, we use the superconducting transition temperature $T_{\text {c}}$ as a comparative metric to parameterize the thin film and link the deposition conditions and transport parameters to the crystalline structure probed by GID. We then use the optimized films to fabricate SNSPDs and evaluate their performance.

### Relation between growth condition, transition temperature $T_{\text {c}}$ and crystallinity

**Fig. 1:**
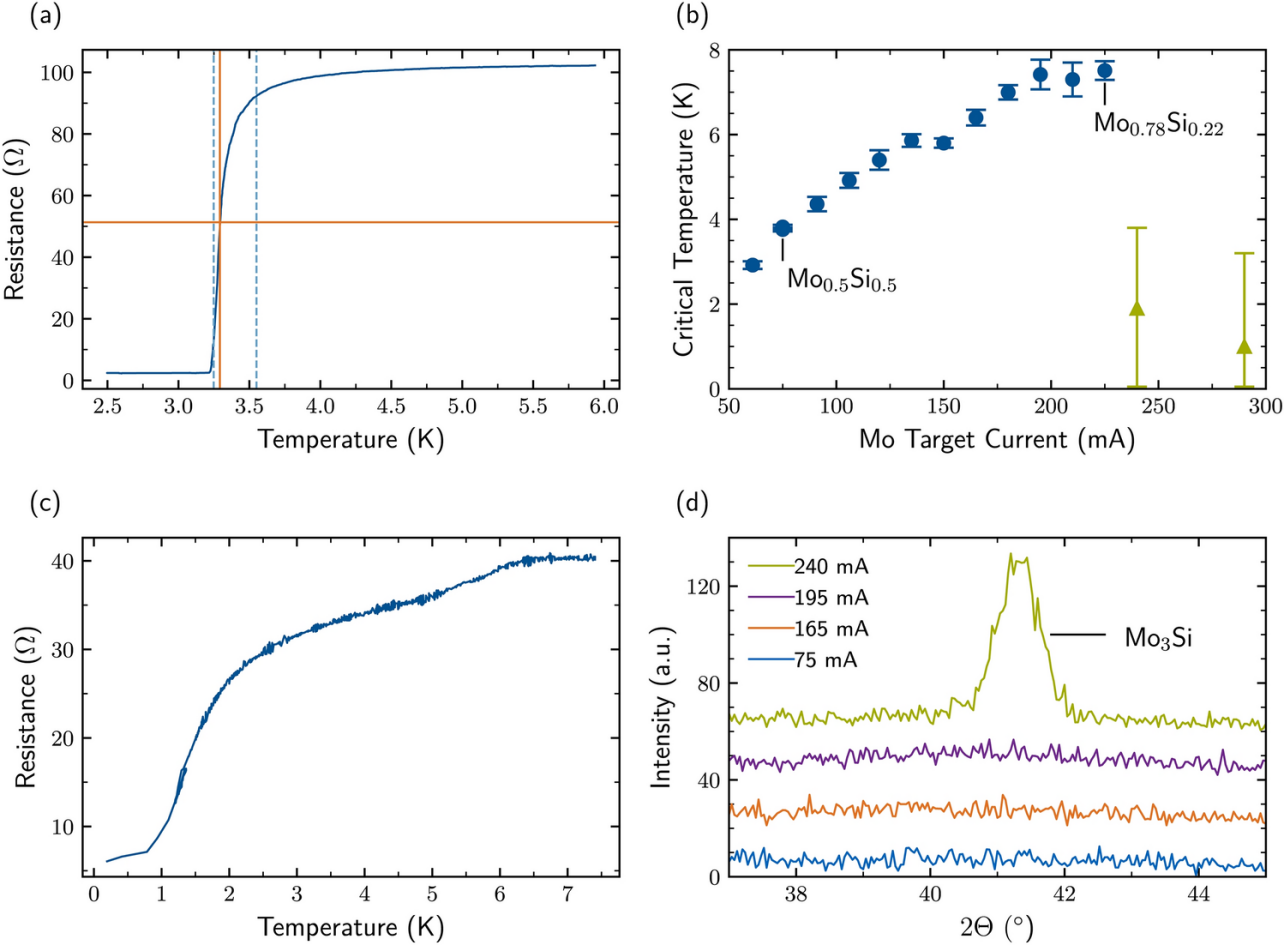
**(a)** Example of a resistive transition and evaluation method for $T_{\text {c}}$ and the transition width. $T_{\text {c}}$ is defined as the midpoint $R(T_{\text {c}})=0.5 \cdot R_{\text {nc}}$, as indicated by the orange lines, where $R_{\text {nc}}$ is the normal conducting resistance. The transition width corresponds to the temperature interval where the resistance is between 10% and 90% of the normal conducting resistance (dashed blue lines). Here, $T_{\text {c}}$ = ${3.29}\,\hbox {K}$ and the transition width is equal to ${0.30}\,\hbox {K}$. The film showing such a transition has a concentration of 50% of Mo. **(b)** $T_{\text {c}}$ of MoSi films as a function of the DC current applied to the Mo target with a fixed power of 75 W to the Si target. The Mo concentration of the films is extracted independently from XPS measurements. We observe an increase in $T_{\text {c}}$ up to a concentration of 78%, above this concentration value the films present a broad transition with lower values of $T_{\text {c}}$. **(c)** Resistance curve of a film deposited with a DC Mo target current of 240 mA resulted in a film with a 81% Mo concentration. The films shows an irregular resistive transition shape and transition width, indicating an inhomogeneity within the film. **(d)** GID spectra for samples with different Mo target DC currents. The films below 195 mA show no presence of any polycrystalline phase, whereas for 240 mA a diffraction peak centered at ${41.2}^{\circ }$ appears, indicating the formation of polycrystalline $\hbox {Mo}_3$Si.

To control the stoichiometry of the superconducting MoSi thin films we vary the DC current or RF power applied to the individual Mo and Si targets, respectively, which changes the deposition rates of the materials. Using a nominal deposition pressure of ${1.2\times 10^{-2}}\,{\textrm{mbar}}$ and a fixed RF power of 75 W applied to the Si sputter target, we tune the DC current applied to the Mo target from 60 mA to 290 mA to vary the nominal film stoichiometry. XPS measurements confirm that this variation results in a change of the relative Mo concentration within the films in the range from 41% to 90%.

In Figure 1a we present typical temperature dependent resistance data from which we determine $T_{\text {c}}$ by measuring the temperature dependent film resistivity. The resistance is measured in a four-probe configuration and $T_{\text {c}}$ is defined as the midpoint of the transition $R(T_{\text {c}})=0.5 \cdot R_{\text {nc}}$ as indicated by the orange lines on the plot, where $R_{\text {nc}}$ is the maximum resistance value. The error bars of the measured $T_{\text {c}}$ values represent the transition width, which marks the temperature interval in which the resistance varies between 10% and 90% of the normal conducting resistance. This value reflects the homogeneity of the deposited film, where an inhomogeneous film will present a wider value due to subsequent transitions^36^. In Figure 1a $T_{\text {c}}$ has a value of ${3.29}\,\hbox {K}$, as indicated by the orange lines while the temperature interval has a value of ${0.30}\,\hbox {K}$ and is marked by the blue dashed lines. Figure 1b shows the $T_{\text {c}}$ dependence on the Mo target current. As can be seen in this figure, we observe a progressive increase of $T_{\text {c}}$ until a Mo concentration of 78%, reaching a $T_{\text {c}}$ of 7.9 K. For a Mo concentration of 81% $T_{\text {c}}$ suddenly drops and shows a large transition width. As presented in Figure 1c for Mo concentrations exceeding 81%, the temperature dependent transport characteristics show a much broader and more irregular incremental transition that differs from the smooth step-like behavior seen in Figure 1c. The sudden step-like change in Figure 1a is indicative of a homogeneous film undergoing a transition to the superconducting phase. In contrast, the transition seen in Figure 1c comes with a broader decrease of the measured $T_{\text {c}}$ values and is indicative of increased inhomogeneity within the film, where parts of the film undergo the transition to the superconducting phase at different temperatures. This behavior suggests that the superconducting domains are separated by non-superconducting regions, which manifest in different transition temperatures^37^.

This conclusion is verified by comparing with GID measurements performed for different values of Mo target current. Typical examples of Mo concentration dependent GID data are presented in Figure 1d, where the data are vertically offset for improved clarity. As expected, the GID data exhibits only a broad background signal for Mo concentrations below 78%, consistent with the presence of the amorphous MoSi phase. However, a diffraction peak emerges at an incident angle of $2 \Theta = 41.2^{\circ }$ for Mo concentrations larger than 81%. The emergence of this peak is concomitant with the change observed in the superconducting transition indicative of a sudden change of the microstructure of the MoSi film. We identify this GID peak to the emergence of the polycrystalline phase of $\hbox {Mo}_3$Si (210), in agreement with the report of Bosworth *et al.*^30^. $\hbox {Mo}_3$Si is an A15 compound which has a $T_{\text {c}}$ < 2 K in its crystalline phase^38^. Exceeding the critical Mo amount and the corresponding critical thickness^39^, the crystalline phases dominate the structure and $T_{\text {c}}$ decreases further, reaching down to 1 K for pure Mo^40^. As presented by Koepke *et al.*^41^ a deposition of Mo at cryogenic temperatures allows for higher $T_{\text {c}}$ values, although the compounds undergo a structural transition at temperatures between 120 K and 190 K. The amorphous phase can be maintained by forming metal-metalloid alloys, with sufficient amount of Si in case of MoSi. Moreover, is has been shown that postprocessing techniques, such as irradiation to induce disorder in the material can be used to restore the superconducting properties^38^.

### Influence of the working pressure on crystalline phase formation

After observing that the microstructure of the film undergoes a sudden change upon varying the Mo:Si stoichiometry, we studied the impact of the pressure of the pure Ar sputtering atmosphere, which gives rise to an effective change of the deposition rate and change in thermalization of the sputtered species at fixed stoichiometry.

**Fig. 2:**
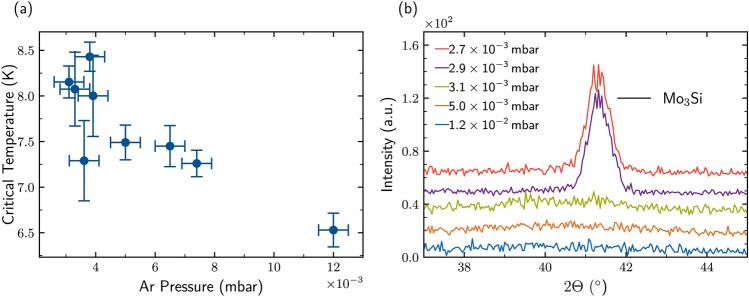
**(a)** Dependence of $T_{\text {c}}$ on the Ar pressure during sputter deposition. A maximum is observed between ${3.2\times 10^{-3}}\,{\textrm{mbar}}$ and ${3.8\times 10^{-3}}\,{\textrm{mbar}}$. For pressures below ${3.0\times 10^{-3}}\,{\textrm{mbar}}$ we observe irregular, step wise resistive transitions, caused by inhomogeneities in the films. **(b)** GID spectra for films deposited at different working pressures. Films deposited with pressures below ${3.0\times 10^{-3}}\,{\textrm{mbar}}$ show a diffraction peak corresponding to $\hbox {Mo}_3$Si (210) in the A15 structure.

In Figure 2a, we present the obtained $T_{\text {c}}$ values measured for a series of deposition runs at working pressures from ${1.2\times 10^{-3}}\,{\textrm{mbar}}$ down to ${2.7\times 10^{-3}}\,{\textrm{mbar}}$, while keeping the applied target power and current constant resulting in a $\hbox {Mo}_{0.71} \hbox {Si}_{0.29}$ film concentration. This stoichiometry was chosen as it provides a high enough centered $T_{\text {c}}$ value to obtain measurable transitions ($>{1}\,\hbox {K}$ limited by the minimum temperature of the cryostat) allowing a full study over the entire pressure range. Furthermore, it is not directly located at the critical concentration to avoid entering the crystalline phase regime for small concentration variations. We identify a trend towards higher $T_{\text {c}}$ values for lower pressures and find an optimum in $T_{\text {c}}$ for deposition pressure between ${3.2\times 10^{-3}}\,{\textrm{mbar}}$ and ${3.8\times 10^{-3}}\,{\textrm{mbar}}$, reaching maximum values of $T_{\text {c}}$ of 8.4 K with a transition width of 0.3 K. On the other hand, no clear $T_{\text {c}}$ value could be determined below ${3.0\times 10^{-3}}\,{\textrm{mbar}}$. For this film, the resistive transition exhibits an incremental transition behaviour similar to the one observed for high Mo concentrations in the previous sub-section.

The influence of the Ar pressure on $T_{\text {c}}$ can be explained by two major effects on the deposition rates. For high pressure values the thermalization of atoms is enhanced, due to an increased scattering rate of the Mo and Si atoms with Ar ions. Thus, the sputtered atoms arrive at the substrate with lower average kinetic energy. Moreover, backscattering effects occur, where the atoms are scattered by the Ar atoms back into the atmosphere. Altogether, this results in a reduction of the deposition yield and, in turn, in an increase of the impurity and defect density in the film^42^. Reducing the Ar pressure improves the film growth in a sense that atoms arrive with higher average kinetic energies at the substrate, as less Ar ions are present for scattering. As a result, atoms loosely bound to the sample surface are pinched off, leading to a more homogeneous film growth. Under these conditions, for pressures above ${3.2\times 10^{-3}}\,{\textrm{mbar}}$, a homogeneous amorphous film is obtained, which is confirmed by the absence of the diffraction peak in GID measurements (Figure 2b). However, deposition at too low pressure and with too high average kinetic energies result in an implantation of atoms, as well as removal and redistribution of already deposited material. As shown by Krause *et al.*^39 ^this results in the build up of compressive stress^43^, favoring the formation of polycrystalline intermetallic compounds such as $\hbox {Mo}_3$Si driven by thermodynamic processes. GID measurements performed on films deposited at pressures below ${3.0\times 10^{-3}}\,{\textrm{mbar}}$ indeed show the resulting $\hbox {Mo}_3$Si diffraction peak. From our study we conclude that the pressure range between ${3.2\times 10^{-3}}\,{\textrm{mbar}}$ and ${3.8\times 10^{-3}}\,{\textrm{mbar}}$ provides the best growth conditions to obtain high $T_{\text {c}}$ values and fully amorphous and homogeneous film structures. We note that a variation of the pressure also has an influence on the deposition rates, and thus on the ratio of the rates. However, this change in rate ratio is not sufficient to explain the trend observed. Moreover, the observed $T_{\text {c}}$ values obtained will be specific to magnetron co-sputtering system used. However, the general observation of a transition from amorphous to polycrystalline film growth is expected to be universal.

### Influence of deposition temperature on crystalline phase formation

**Fig. 3:**
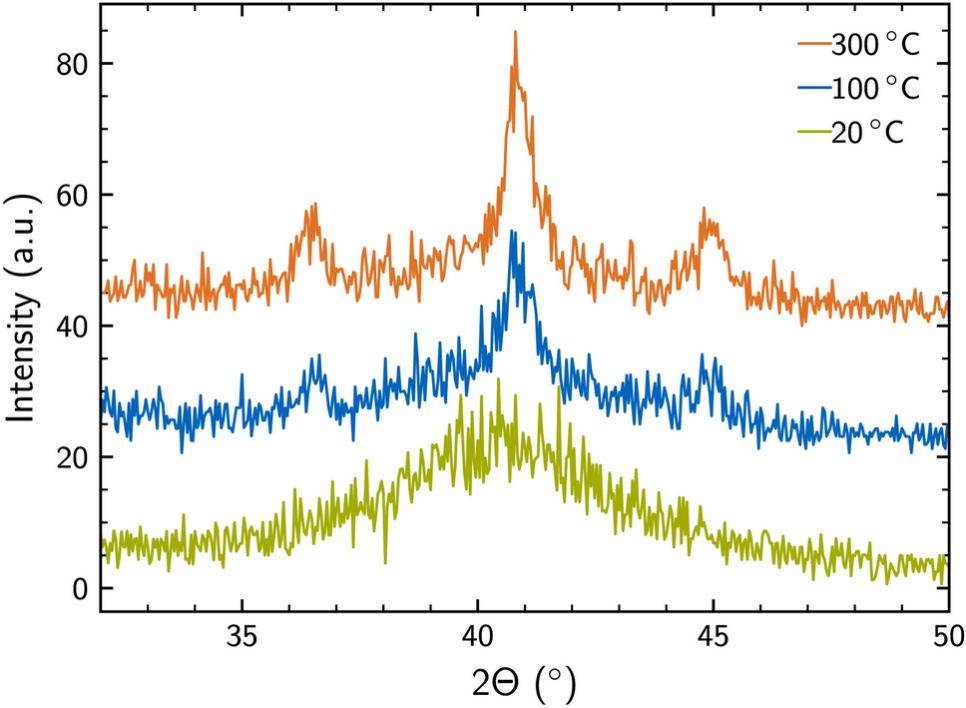
GID data recorded from MoSi films deposited at different temperatures. Films deposited at temperatures above room temperature show a dominant formation of crystallites with the (210) planes parallel to the substrate surface. Additional orientations such as the (200) and (211), as well as (103) are present.

We continue to investigate the influence of the deposition temperature on the resulting thin film properties. To this end, we sputter $\hbox {Mo}_{0.71}\hbox {Si}_{0.29}$ films at a chamber pressure of ${3.1\times 10^{\mathrm{-3}}}\,{\textrm{mbar}}$. We explored deposition temperatures ranging from ${{20}\,^{\circ } \text {C}}$, to ${{100}\,^{\circ } \text {C}}$ and ${{300}\,^{\circ } \text {C}}$. Figure 3 presents the GID data obtained for these films in the region of interest around the expected (210) diffraction peak of $\hbox {Mo}_3$Si for different deposition runs.

As can be seen in Figure 3, for room temperature deposition, a broad signal and no clear diffraction peak is visible, indicating an amorphous or polycrystalline film without any texture. The values for $T_{\text {c}}$ are in a range of $7.8 - {8.5}\,\hbox {K}$ with a mean transition width of 0.7 K, but no clear trend is observed. The data obtained at elevated deposition temperatures show more pronounced diffraction peaks and significantly reduced full-width at half-maximum (FWHM) indicative of increasing formation of crystallites with a preferred orientation. For the highest deposition temperature of ${{300}^{\circ } \text {C}}$ we observe the (200) at $2 \Theta = 36.5^{\circ }$ and (211) centered around $2 \Theta = 45.4^{\circ }$ orientations. In addition, the right hand side peak overlaps with $\hbox {MoSi}_2$ in the (103) orientation $2 \Theta = 44.6^{\circ }$, which could also be present. We note that in the study of the temperature dependence we increased the thickness to 60 nm to increase the signal intensity in the GID spectra. As a result, we find a finite texture only for the thicker films, whereas no evidence of any texturing was found for film thicknesses below 10 nm. Evidently, the formation of a textured film is absent at this stoichiometry for a thickness below 11.3 nm^39^. From these observations we conclude that even though the films show a superconducting transition, higher deposition temperatures enhance the formation of the crystalline phase. Hence, deposition at room temperature enhances the amorphousivity of the film.

### Single-Photon detection with high $T_{\text {c}}$ thin films

Finally, we fabricated SNSPDs using the optimized $\hbox {Mo}_{0.71}\hbox {Si}_{0.29}$ thin films having a film thickness of 4.3 nm covered with a Si capping with a thickness of 4 nm to avoid oxidation. The meander type detectors cover an area of $10 \times {10}\,{\upmu {\textrm{m}}}^{2}$ and have a defined wire width of 100 nm. The thin film from which the detector was fabricated has a sheet resistance measured at 20 K of around ${350}\,{\Omega {\Box }^{-1}}$ and a transition temperature $T_{\text {c}}$ of 6.2 K.

**Fig. 4:**
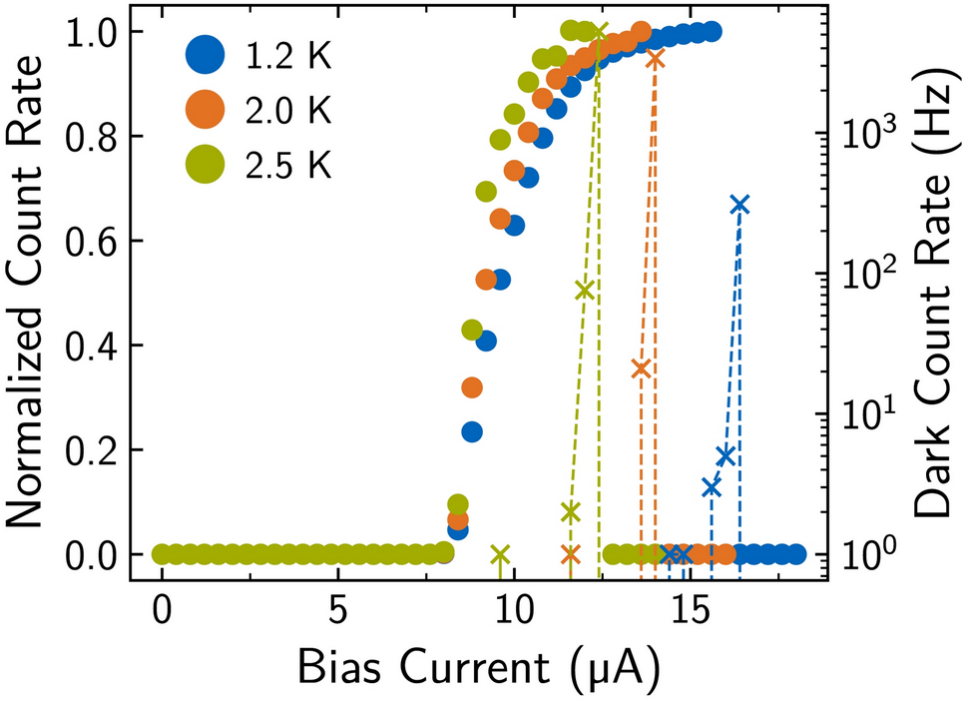
Normalized count rates of a SNSPD illuminated with 780 nm laser light operated at different temperatures. The count rate is saturating towards unity with the bias current approaching the critical current of the detector, which is shifted to higher values from ${12.4}\,{\upmu {\textrm{A}}}$ to ${16}\,{\upmu {\textrm{A}}}$ for lower operating temperatures.

Upon photon absorption a normal-conducting hotspot is generated locally, deflecting the current into the side channels between edge of the nanowire and hotspot. This leads to the formation of a resistive barrier across the entire cross section of a nanowire, which can be measured as a voltage pulse^44^. For the application as single-photon detectors there is a trade off between operational temperatures, which limits the suitable setups and a small superconducting energy gap, which is related to low $T_{\text {c}}$ values and impacts the sensitivity^19^.

Figure 4 shows typical data for the count rate and dark count rate as a function of the applied bias current. Here, the same detector is characterized at three different temperatures in the range between 1.2 K and 2.5 K and homogeneously flood illuminated with a 780 nm laser. The count rates are normalized to their saturation value. This plateau region indicates a unitary internal detection efficiency, or quantum efficiency, where every absorbed photon generates a voltage pulse. We find a systematic increase of the plateau width with decreasing operating temperature, which originates from the increase in switching current towards lower temperatures from ${12.4}\,{\upmu {\textrm{A}}}$ at 2.5 K to above ${15}\,{\upmu {\textrm{A}}}$ at 1.2 K. At the same time, the dark count rate, which increases exponentially for bias levels towards the switching current, reaches lower absolute values at lower temperatures. For optimal operation, the detector should be biased at a current value where the count rate curve saturates and where the detector is fully sensitive, exhibiting a dark count rate below 1 Hz. With the optimized MoSi film we achieve saturating internal quantum efficiency allowing operation at elevated temperatures of 2.5 K, where standard closed cycle cryogenic systems can still be used. We note here that the wire width of 100 nm could be further reduced in order to achieve saturating detection efficiency also for longer wavelengths, where the photon energy is lower, and the corresponding hotspot size is smaller.

## Conclusion

In summary, our results demonstrate the ability to tailor the transition temperature $T_{\text {c}}$ of MoSi thin films by controlling the Mo:Si stoichiometry and growth conditions. The observed values of $T_{\text {c}}$ increased up to a Mo concentration of 78% in the compound, reaching maximum values of 8.4(3) K for a film thickness of 17.7(8) nm. Furthermore, we investigated the presence of crystalline phases and observed an increased volume fraction of the polycrystalline phase $\hbox {Mo}_3$Si for Mo concentrations of 81% and higher. Moreover, we found that the growth conditions, such as the chamber pressure and the deposition temperature, influence the structural growth of the thin films. The presence of the polycrystalline phase results in an enhanced inhomogeneity in the film and leads to a significant decrease in $T_{\text {c}}$. In order to deposit thin films optimized for SNSPD fabrication, the amorphous phase should be maintained, which is ensured by choosing Mo concentrations below 78% and choosing a deposition working pressure between ${3.2\times 10^{-3}}\,{\textrm{mbar}}$ and ${3.8\times 10^{-3}}\,{\textrm{mbar}}$. Knowledge of these growth conditions and the identification of a sudden morphological phase transition will allow the optimized growth of MoSi thin films in different growth systems. The characterization of our SNSPDs at 780 nm showed near unity internal detection efficiency at an operation temperature of 2.5 K, and saturating behaviour for lower operational temperatures while maintaining a dark count rate below 1 Hz.

## Data Availability

The datasets generated during and/or analysed during the current study are available from the corresponding authors on reasonable request.
